# Evidence of early genomic selection in Holstein Friesian across African and European ecosystems

**DOI:** 10.1186/s12864-025-11828-y

**Published:** 2025-07-01

**Authors:** Junxin Gao, Rayner Gonzalez-Prendes, Ying Liu, Juha Kantanen, Catarina Ginja, Nasser Ghanem, Donald Rugira Kugonza, Mahlako Makgahlela, Henk Bovenhuis, Martien A.M. Groenen, Richard P.M.A. Crooijmans

**Affiliations:** 1 Animal Breeding and Genomics, Wageningen University & Research, Wageningen, The Netherlands; 2 Natural Resources Institute Finland, Jokioinen, Finland; 3 Faculty of Veterinary Medicine, CIISA, University of Lisbon, Lisbon, Portugal; 4 Animal Production Department, Faculty of Agriculture, Cairo University, Giza, Egypt; 5 Department of Agricultural Production, College of Agricultural and Environmental Sciences, Makerere University, Kampala, Uganda; 6 Agricultural Research Council-Animal Production Institute, Irene, South Africa; 7 Department of Animal, Wildlife and Grassland Sciences, University of the Free State, Bloemfontein, South Africa

**Keywords:** Holstein Friesian, Dairy farming, Whole-genome resequencing, Adaptation, Selective sweeps

## Abstract

**Background:**

The Holstein Friesian (HF) cattle breed is the most dominant breed in commercial dairy farming worldwide and managed in more than 150 countries. These countries span diverse agro-climatic zones, ranging from tropical to cold regions. The introduction of HF animals in these regions occurred at different moments in the past which are poorly recorded and continued through importation of live animal and frozen semen. We hypothesize that the HF cattle populations in these regions underwent early forms of adaptation to these specific local environments. However, the detection of genetic variation associated with this adaptation remains poorly documented.

**Results:**

This study investigates genetic relationship and potential early selection signatures in HF populations from three African countries (Egypt, South Africa, Uganda) and three European countries (Finland, Portugal, The Netherlands), considering five animals per country. Approximately 16.0 million single nucleotide polymorphisms (SNPs) were detected in the 30 HF animals and used for further analyses.

Across all countries, we identified dispersed regions totaling 3.3 megabase of ecosystem-specific genomic regions (43 genes), indicative of early selection signatures based on fixation indices (*F*-statistic, *F*st). Furthermore, comparing variants between tropical (Egypt and Uganda) and cold regions (Finland and The Netherlands) by *F*st, nucleotide diversity (*θ*_π_ ratio), and extended haplotype homozygosity (XP-EHH), we identified a total of 10 candidate regions, comprising 12 genes within a 0.57 megabase size. The regions were enriched with genes involved in signaling pathways associated directly or indirectly with adaptation, including the immune system (*PGLYRP4*,*PGLYRP3*, *PAG1*, *CD48*, *SLAMF1*,* DYSF*,and *LOC615223*), organ development and reproduction (*LDB3*, *ADAMTSL4*, *TPRN*, *CCDC40*, *OR2AG1G*, and *OR8B3*), thermogenic activation (*TBC1D16*), phospholipid metabolism (*PLPPR4* and *PITPNB*), thermos-tolerance (*ZNF423*), and stimulus response (*NCOA7*, *CYP2C85*, and* ARFGEF3*).

**Conclusion:**

This study provides new insights into early forms of genetic plasticity of animals adapted to very diverse ecosystems. Our findings highlight candidate genes related to immune response, organ development, reproduction, metabolism, and thermo-tolerance, hypothesizing their role in facilitating adaptation to different environments.

**Supplementary Information:**

The online version contains supplementary material available at 10.1186/s12864-025-11828-y.

## Background

The Holstein-Friesian (HF) breed, classified under *Bos taurus*, is a dominant cattle breed in dairy farming. Its lineage can be traced back for over two millennia to Frisia, which covers the current regions of North Holland, Friesland, and Groningen, and extends beyond the German border to the Ems River [1]. Owning to their highly valued milk production capabilities, HF cattle were initially imported into the United States in 1852, eventually achieving a global distribution [2]. Despite the extensive movement of HF cattle, the precise time points of their introduction, subsequent reintroductions, and the global trade of frozen semen are sporadically documented in the literature, though some evidence suggests a global movement dating back approximately a century [3, 4]. For instance, historical records indicate that HF cattle were predominantly introduced into Finland in the mid-20th century through the use of semen from artificially inseminated (AI) bulls for crossbreeding with indigenous cattle breeds [5]. During the same period, HF cattle were crossbred with local Dutch Friesians in The Netherlands using both live cattle and frozen semen [6]. The introduction of HF cattle into Portugal occurred mainly in the 20th century, facilitated by the importation of live animals [7], although historical records indicate the so called “*Turina*” cattle exist in Portugal since 1758 [8]. The 1925 census reported about 15,000 HF animals in Portugal [9]. Furthermore, HF cattle were introduced into vastly different environments from their European origins, such as the sub-/tropical climates of Africa in the early twentieth century through both live cattle and frozen semen [10–12]. In addition to varying environmental conditions, persistent exposure to multiple stresses of feed quality, high disease and parasitic incidences, and diverse husbandry and breeding practices have also contributed to the divergence of cattle ecosystems [13]. Therefore, these factors likely facilitated the accumulation of early forms of locally adaptive traits within HF cattle populations genetic variations in morphological and physiological adaptations, including but not limited to metabolic, skin pigmentation, immunity, and heat tolerance [14, 15].

Under strong positive selection, advantageous heritable traits become more prevalent, while detrimental traits diminish over generations [16]. In cattle, some selection has led to the development of divergent breeds specialized for milk, beef, or dual purposes [17]. Other selection strategies focus on adaptive characteristics like disease resistance or immune responses [18, 19]. At the genetic level, standard selection models involve loci with two alleles, where one allele is favored over the other so that its frequency may increase until it reaches fixation [20, 21]. For instance, intensive artificial selective in dairy cattle has led to a shift in allele frequency at the *DGAT1* gene for desirable milk production traits [22]. This frequency dynamic in the genomic region can produce “selection signatures”, where the hitch-hiking effect reveals that changes in allele frequency at a particular locus per generation are not only influenced by the allele itself, but rather because it is closely linked to a target gene undergoing strong selection [23]. Selective sweep, on the other hand, is a specific type of selection signature. A selection sweep occurs when a beneficial mutation arises and increases in frequency within a population. This process reduces nucleotide diversity in the surrounding region of the genome due to genetic hitchhiking. The hitchhiking haplotype is expected to be relatively long because of strong linkage disequilibrium (LD) [24].

Across different cattle breeds and species, selection signatures and selective sweeps have been identified and associated with adaptive traits influenced by human and climate-mediated migration. These traits include immune response, reproductive processes, metabolic processes, and heat tolerance [17]. Additionally, selection signatures have been linked to commercial traits such as milk production, reproduction, and muscle formation [15, 25, 26]. However, research on genomic markers specific to HF cattle within different ecosystems remains scarce. Despite the extremely high milk production of HF cattle in various countries with different ecosystems [27], HF cattle exhibit poor tolerance to heat and diseases compared to natural breeds, especially in tropical agro-climatic areas [28, 29]. This susceptibility leads to reduced production and health capacity under such conditions [22]. Investigating the genomes of HF cattle to understand how they adapt to diverse ecosystems would be beneficial not only in tropical regions but also in temperate countries facing changing climate conditions.

Therefore, this study aims to explore the unique genetic characteristics of HF cattle genomes from different ecosystems. We present a comprehensive genomic analysis of the variations in the nuclear genomes of 30 HF cattle sampled from three African and three European countries (i.e. across north through southern latitudes). Our findings provide insights into the early selection in different ecosystems that might help HF cattle adapt to diverse environments.

## Results

### SNP distribution among Holstein Friesian cattle from various countries

**Fig. 1:**
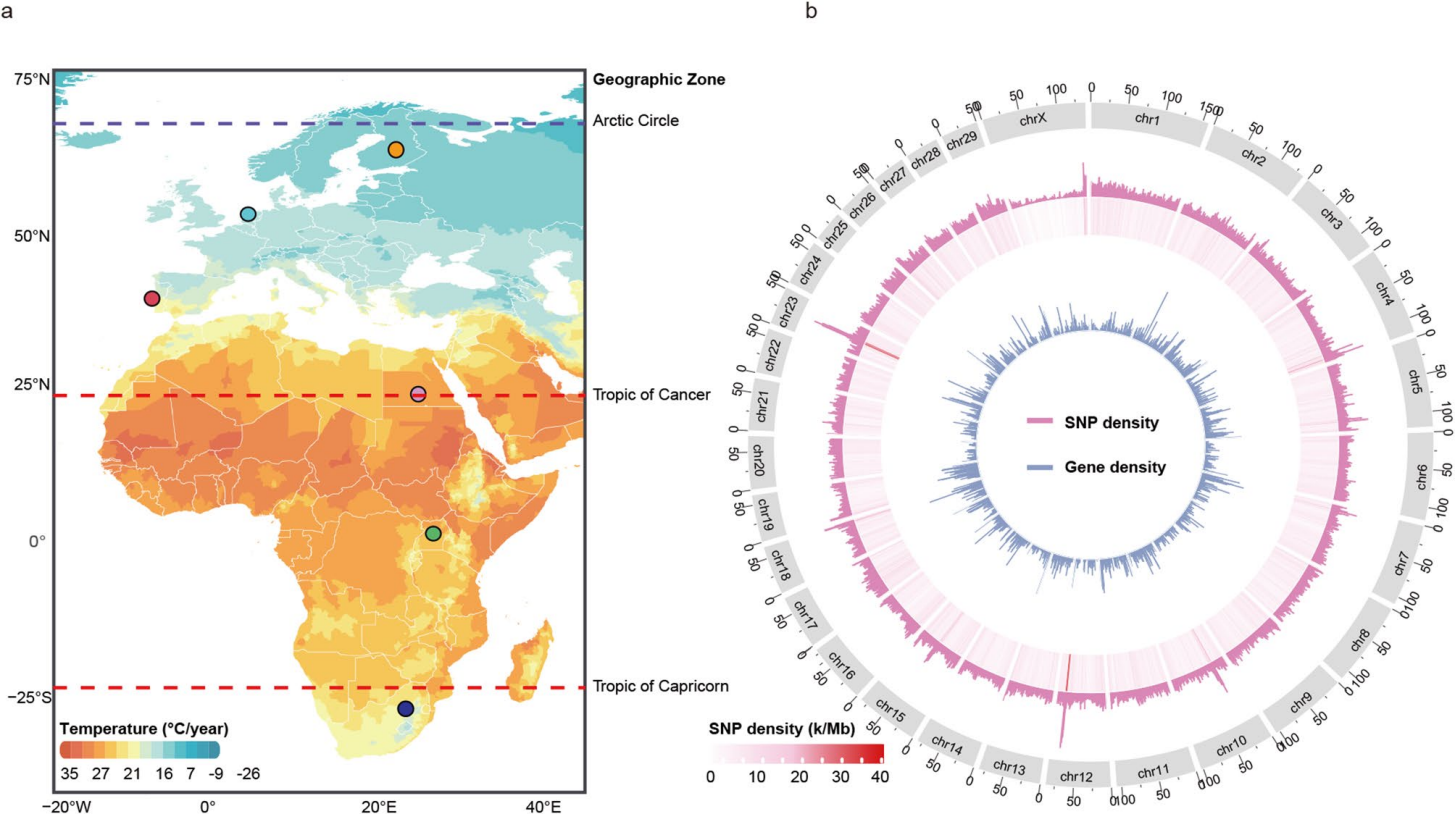
HF sampling and SNPs distribution from various countries. **a** Geographic location of 30 HF included in this study. The map images were created by authors using https://impactlab.org/map and GPS data in R package: maps. Sampling countries included Egypt (*n* = 5), Finland (*n* = 5), Portugal (*n* = 5), South Africa (*n* = 5), The Netherlands (*n* = 5), and Uganda (*n* = 5). **b** Chromosome-wise SNP distribution heat map across HF cattle genomes. The horizontal axis shows the chromosome length across 1 Mb windows; The pink heat map and bar plot show the chromosome density (SNP count/kb). The blue bar plot represents the calculation of gene density (genes count/Mb) across 1 Mb windows

A total of 30 HF individuals from various geographic zones were sampled for this study. These cows were chosen from three African (Egypt, South Africa, Uganda) and three European countries (Finland, Portugal, The Netherlands) (Fig. 1a). The extracted DNA was whole genome re-sequenced, producing approximately 493 Gigabyte (Gb) of Illumina short-read data at an average coverage of 10.2× (Supplementary material 1).

SNP calling against the ARS-UCD1.2 reference genome originally identified a total of 17.5 million variants, including ~ 15.6 million single-nucleotide polymorphisms (SNPs) (Table S1, Supplementary material 2). After filtering based on missing genotypes and minor allele frequency (removing any SNPs with a missing rate > 5% or MAF < 5%), about 11.9 million high-quality SNPs were retained (Table S2, Supplementary material 2). Annotation information based on their genomic locations and the impact of amino acid substitutions on protein function (sorting intolerant from tolerant algorithm, SIFT) are provided in Table S3-S5, Supplementary material 2.

These high-quality SNPs were evenly distributed across the whole genome (Fig. 1b, Table S2). A high density of SNPs was observed on *Bos taurus* autosome 23 (BTA23) between 25 and 30 Megabase (Mb), which harbours the Major Histocompatibility Complex (MHC), also known as the Bovine Leukocyte Antigen (*BoLA*) Complex [30]. Another outlier region was located on BTA12 between 70 Mb and 73 Mb, which contains genes related to the ATP-binding cassette (ABC) family. This region encodes ABCG4 proteins that transport various xenobiotics across the plasma membrane and cholesterol into milk [31].

### Genetic relationship among Holstein Friesian cattle populations

**Fig. 2:**
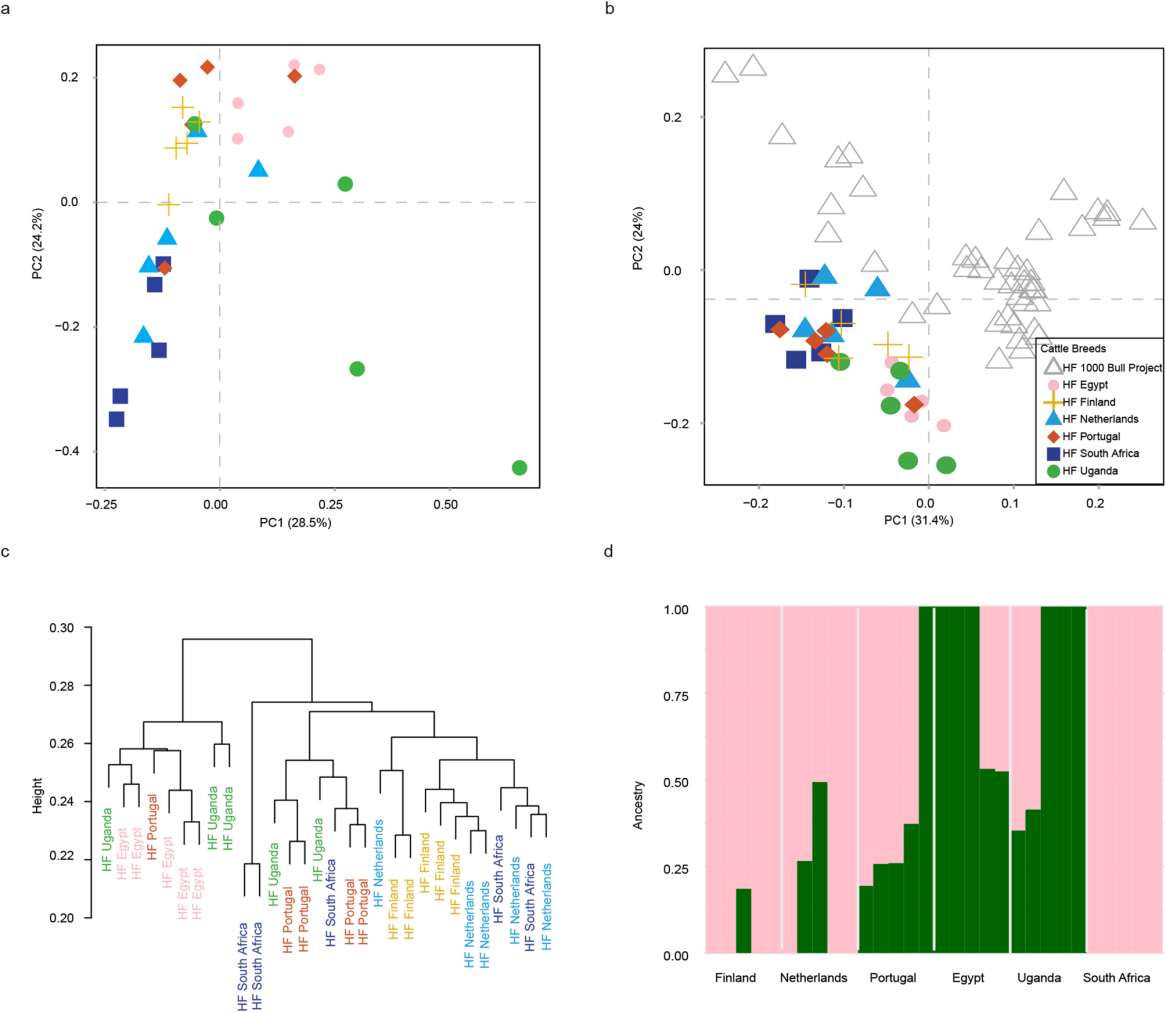
Genetic structure of HF across different countries. **a** PCA showing PC1 versus PC2 of 30 HF across six countries. **b** PCA showing PC1 versus PC2 of 30 HF and 42 previously published HF genomes. **c** Hierarchical clustering (Ward method) on the distance matrix based on autosomal SNP data (*n* = 72). **d** Admixture patterns of 30 HF for *K* = 2

To explore the genetic relationships among 30 HF genomes from six countries, the final data included approximately 11.6 million autosomal SNPs. Principal component analysis (PCA) of these SNPs revealed slight geographic differentiation among HF populations from different countries along PC1 and PC2 (Fig. 2a). Hierarchical clustering based on the distance matrix also delineated distinct geographic groups of HF (Fig. 2c). HF cattle from tropical regions (Uganda and Egypt) were relatively distant from those in Finland and The Netherlands (Fig. 2c). HF cattle from Portugal and South Africa occupied genetically intermediate clusters between those of Finland/The Netherlands and Egypt/Uganda. The ADMIXTURE analysis recovered a similar pattern and identified small differences between HF groups from Finland/South Africa/The Netherlands and Egypt/Portugal/Uganda at *K* = 2 (Fig. 2d).

Even through the most recent generation of HF are local HF, we cannot exclude the possibility of early admixture events. To further elucidate the genetic background of HF cattle, 30 HF genomes were combined with 42 previously published HF genomes from the 1000 bull project [32]. This combined dataset yielded approximately 13.2 million autosomal SNPs (Supplementary material 3). The PCA plot of the combined SNPs revealed unclear differentiation, with PC1 indicating that 30 HF fell in an intermediate position within the published HF cattle (Fig. 2b). The optimal cross-validation error (CV) at *K* = 1 from ADMIXTURE analysis also indicated a low possibility of subpopulations (Figure S1, Supplementary material 4).

### Allele frequency divergence of SNPs within country populations

Selection is a non-random process often influenced by survival differences in specific environments [33]. Economic heritable traits become more prevalent over generations, while detrimental traits tend to diminish. HF cattle were introduced into various countries, where they may have been subjected to both artificial and natural selection. It is hypothesized that SNPs with significantly different allele frequencies between HF populations in different countries have been influenced by specific selection pressures [34].

**Fig. 3:**
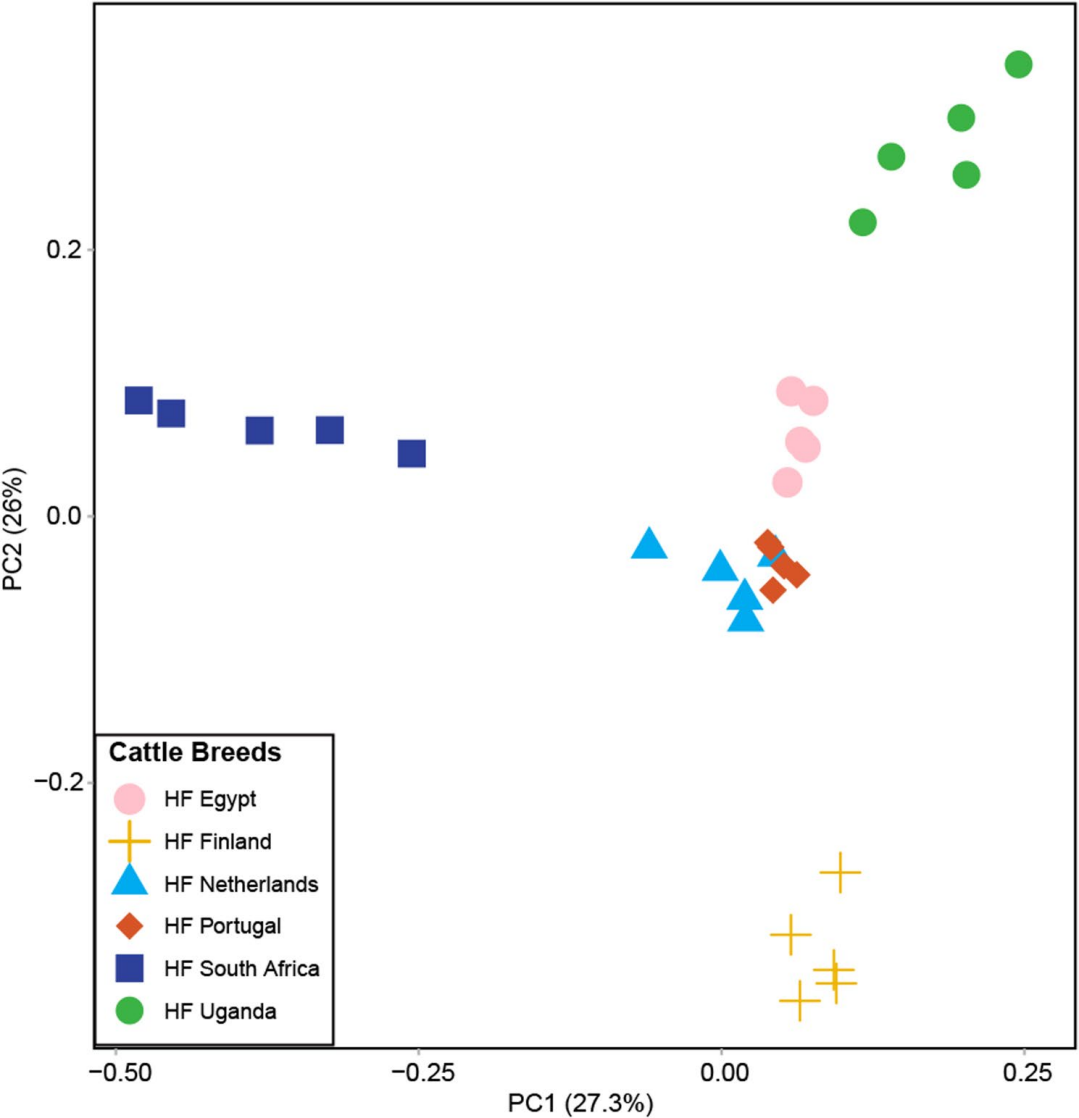
PCA depicting the genetic structure of potential selected alleles of SNPs between different countries

To identify potential selective alleles of SNPs across HF populations from six countries, we established a cutoff: an allele frequency of ≥ 0.6 in one country and < 0.3 in the remaining five. This threshold helps pinpoint SNPs that are potentially advantageous in specific environments. We identified 31,753 SNPs as potentially selected, including 100 SNPs that were nonsynonymous (causing stop-gained or missense mutations) (Table 1). These SNPs overlapped with 78 genes (Table 1). Detailed annotation of these SNPs and genes was performed using the Animal QTL Database (QTL db) and Gene Ontology (GO) categories (Supplementary material 5). To further explore the structure of these potential selected alleles, we performed a PCA on these 31,753 SNPs (Fig. 3). The PCA revealed distinct separations along PC1/PC2 among HF populations from the six countries. Dutch and Portuguese HF populations exhibited a close genetic relationship, while populations from Finland, Uganda, and South Africa appeared genetically distinct from each other. This genetic divergence is likely due to the unique selection pressures in each country and the adaptive retention of the national HF populations.

**Table 1:** Summary of SNPs with potential selective alleles

HF populations	Uganda	Finland	Egypt	South Africa	The Netherlands	Portugal
Count of SNPs	4,067	5,634	4,522	8,437	3,442	5,651
Count of nonsynonymous SNPs	17	12	11	29	12	19
Overlapped genes (nonsynonymous SNPs)	12	10	9	26	12	9

### Genome-wide selection signatures for early adaptation within countries

To study the early adaptation of HF populations within countries, we performed a signature of selection analysis. One well-established method to detect selection signatures is using fixation indices (*F*-statistic, *F*st) [35]. The *F*st approach quantifies population differentiation based on locus-specific allele frequencies, revealing genomic regions with highly differentiated alleles that are undergoing divergent selection. Despite limited unrelated sample sizes (*n* = 5 per country), estimates of genetic differentiation measured by *F*st remain relatively reliable when using a large number of SNP markers (> 1,000) [36].

**Fig. 4:**
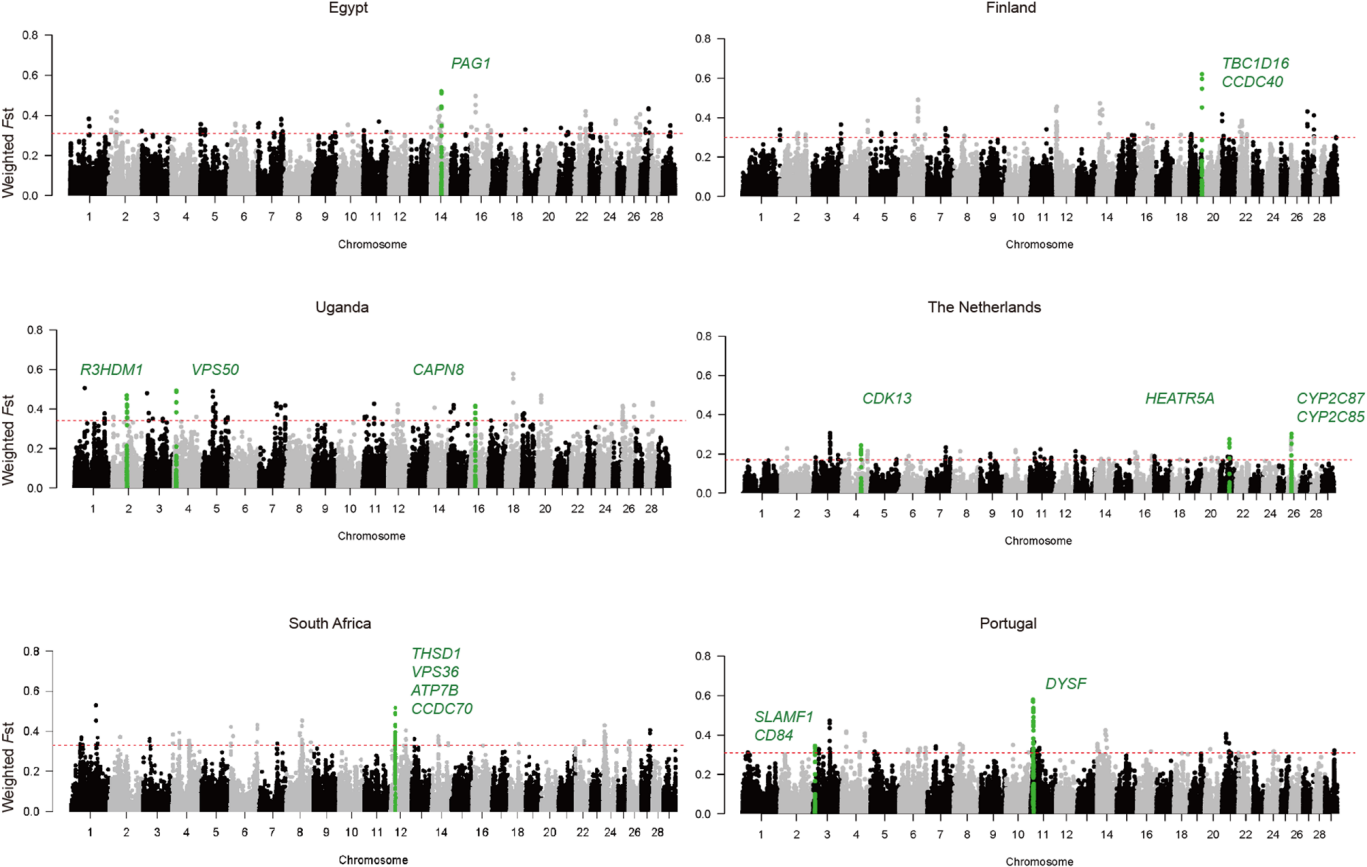
Genome-wide selection signatures observed in each country. Manhattan plots depicting the *F*st values (y-axis) in windows of 50 kb using a 25 kb slide across all autosomes (x-axis). Names of genes with protein-coding variants were highlighted. Using the top 0.1% of *F*st values as significant outliers

In our genome-wide analysis, we compared each country’s population separately against the remaining five countries. Using the top 0.1% of *F*st values [37], we identified potential regions and candidate genes on the autosomes (Fig. 4). A total of 3.3 Mb outlier regions with 43 candidate genes and QTL associated traits were identified from six countries (Supplementary material 6). Seventeen candidate genes with protein-coding SNP types for each country were highlighted in Fig. 4 and presented in Table 2. Enriched QTL db traits and categories from milk, health, and reproduction shown on Supplementary material 7. Notably, the analysis for The Netherlands exhibited relatively low *F*st values (threshold = 0.17, other = 0.32 ± 0.02), indicating low genetic differentiation compared to other countries.

**Table 2:** Summary of candidate genes on selection signatures within countries

BTA	Region (Mb)	Country	Fst (Weighted)	Candidate genes	Annotation(GO)	QTL db	Gene coding SNP Type
14	44.17–44.32	Egypt	0.44–0.52	*PAG1*	Adaptive immune response	Body temperature; Percentage abnormal sperm; Milk yield	Synonymous variant Missense variant
19	52.52–52.57	Finland	0.45	*CCDC40*	Heart looping	-	Synonymous variant Missense variant
19	52.52–52.65	Finland	0.55–0.62	*TBC1D16*	Regulation of receptor recycling	Average daily gain	Synonymous variant Missense variant
12	21.32–21.38	South Africa	0.40	*ATP7B*	Cation transport	-	Synonymous variant Missense variant
12	21.62–21.68	South Africa	0.48	*VPS36*	Protein catabolic process	-	Missense variant
12	21.27–21.33	South Africa	0.37	*CCDC70*	Plasma membrane	-	Synonymous variant Missense variant
12	21.64–21.69	South Africa	0.49	*THSD1*	Focal adhesion assembly	-	Synonymous variant Missense variant
12	21.90–22.00	South Africa	0.43–0.52	*FOXO1*	Metabolic activity	-	Synonymous variant Missense variant
3	8.97–9.01	Portugal	0.31	*SLAMF1*	Inflammatory response	-	Missense variant
3	9.07–9.13	Portugal	0.34	*CD84*	Immune system	-	Missense variant
11	12.83–12.90	Portugal	0.49–0.53	*DYSF*	Macrophage activation	Stature; Body depth; Rump width; Strength; PTA type; Somatic cell score	Synonymous variant Missense variant
2	61.65–61.78	Uganda	0.39–0.42	*R3HDM1*	Nucleic acid binding	-	Synonymous variant Missense variant
4	10.65–10.74	Uganda	0.43–0.49	*VPS50*	Recycling endosome	-	Synonymous variant Missense variant
16	27.00–27.05	Uganda	0.38	*CAPN8*	Calcium ion binding	Sperm motility	Synonymous variant Missense variant
4	80.95–81.08	The Netherlands	0.20–0.22	*CDK13*	Stem cell differentiation	-	Synonymous variant Missense variant
21	41.85–41.95	The Netherlands	0.17–0.27	*HEATR5A*	-^a^	-	Synonymous variant Missense variant
26	16.20–16.22	The Netherlands	0.19	*CYP2C87*	Oxidoreductase activity	-	Synonymous variant Missense variant
26	16.26–16.31	The Netherlands	0.25–0.30	*CYP2C85*	Oxidoreductase activity	-	Synonymous variant Missense variant

^a^Dashes (-) in “Association (GO)” indicate that no GO/QTL db have been associated within the gene

### Genome-wide selective sweeps for early adaptation to tropical regions

Through the importation across geographic zones, genomic regions may have undergone early selection in response to the distinct ecosystem pressures. We therefore performed a comparative analysis for positive selection signatures between samples from tropical zones (Uganda and Egypt) and colder maritime and continental climate zones (Finland and The Netherlands) (Fig. 1a). Selective sweeps are characterized by allele frequency differentiation, reduced genetic diversity, and increased haplotype homozygosity across generations. To detect these sweeps, we integrated three approaches: *F*st, nucleotide diversity (*θ*_π_ ratio), and cross-population extended haplotype homozygosity (XP-EHH) [15].

Three methods integrated outlier signals (top 1% for *F*st and *θ*_π_ ratio; XP-EHH > 2) in overlapping regions, which were considered candidate selective sweeps. A total of 466 (*F*st), 625 (*θ*_π_ ratio), and 122 (XP-EHH) genes in tropical HF cattle were identified, 4 of which overlapped (Table 3, Supplementary material 8). An additional 8 overlapping genes (top 1% for *F*st; XP-EHH > 2) with relatively low reduced genetic diversity are presented in Table 3. 

**Table 3:** Summary of candidate genes in selective sweeps between tropical and colder zones

BTA	Region (Mb)	Fst (Weighted)	θ_π_ ratio	XP-EHH	Candidate gene	Association (GO)	QTL db	SNP Type
3	43.90- 43.93	0.37	4.50	2.79	*PLPPR4*	Phospholipid metabolic process	-	Up/downstream gene variant 3 prime UTR variant Synonymous variant Intron variant
4	12.30–12.35	0.20	3.75	2.72	*PPP1R9A*	Cytoskeleton	Longissimus muscle area	Up/downstream gene variant
5	73.95–73.98	0.20	3.19	2.53	*RBFOX2*	Neuromuscular process controlling balance	-	Up/downstream gene variant Intron variant
9	25.45–25.48	0.31	3.00	2.04	*NCOA7*	Response to oxidative stress	Somatic cell score	Intron variant Up/downstream gene variant
9	76.1–76.12	0.24	2.97	2.13	*ARFGEF3*	Regulation of ARF protein signal transduction	Milk yield	Intron variant Synonymous variant Missense variant
10	77.60–77.63	0.20	3.25	2.72	*FUT8*	Protein glycosylation	Lean meat yield	Up/downstream gene variant
17	67.27–67.30	0.33	4.6	2.10	*PITPNB*	Phospholipid transporter activity	-	Intron variant Up/downstream gene variant
17	67.30–67.34	0.20	4.8	2.14	*ENSBTAG00000052490*	-^a^	Marbling score (Meat)	Up/downstream gene variant
17	67.34–67.35	0.20	4.8	2.14	*ENSBTAG00000049340*	-	-	Up/downstream gene variant
17	68.50–68.53	0.20	3.41	2.01	*EMID1*	Endoplasmic reticulum	Non-return rate (Reproduction)	Up/downstream gene variant 3/5 prime UTR variant Synonymous variant Missense variant Intron variant
18	18.32–18.33	0.27	3.22	3.21	*ZNF423*	Negative regulation of cold-induced thermogenesis	-	Intron variant Up/downstream gene variant
26	16.25–16.27	0.38	3.41	2.34	*CYP2C85*	Xenobiotic metabolic process	-	Up/downstream gene variant Synonymous variant Intron variant

^a^Dashes (–) in “Association (GO)” indicate that no GO/QTL db have been associated within the gene

**Fig. 5:**
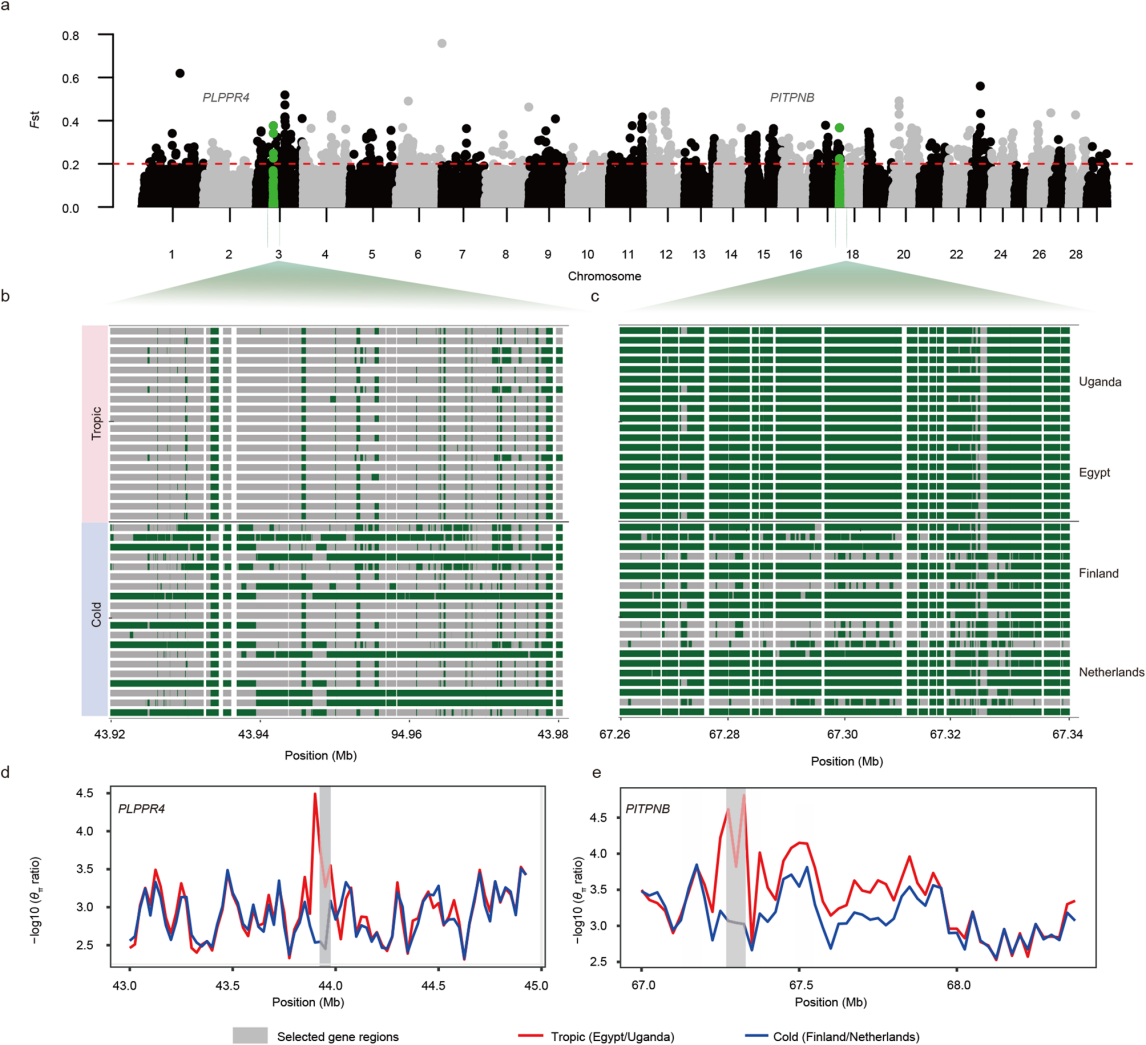
Selective sweeps within the HF tropical cattle. **a** Manhattan plots depicting the *F*st values (y-axis) in windows of non-overlapped 25 kb slides across all autosomes (x-axis). Names of genes with protein-coding variants were highlighted. The red line indicates the significant threshold: top 1% for *F*st (0.20). **b** and **c** Haplotype structures of the overlapped selective sweeps (candidate genes: *PLPPR4*: 43.92–43.98 Mb; *PITPNB*: 67.23–67.34 Mb) on BTA3 and BTA17. Rows correspond to individual animals, while columns represent polymorphic positions in the taurine cattle reference genome. The green color indicates presence of the reference allele, while grey represents the alternative allele. **d** and **e***θ*_π_ ratio analyses of the most significant sweeps and surrounding regions on *PLPPR4* and *PITPNB* genes, comparing tropical (Uganda and Egypt) and colder (Finland and The Netherlands) zones by a 25 kb sliding window. The grey color highlights the regions of candidate genes

**Fig. 6:**
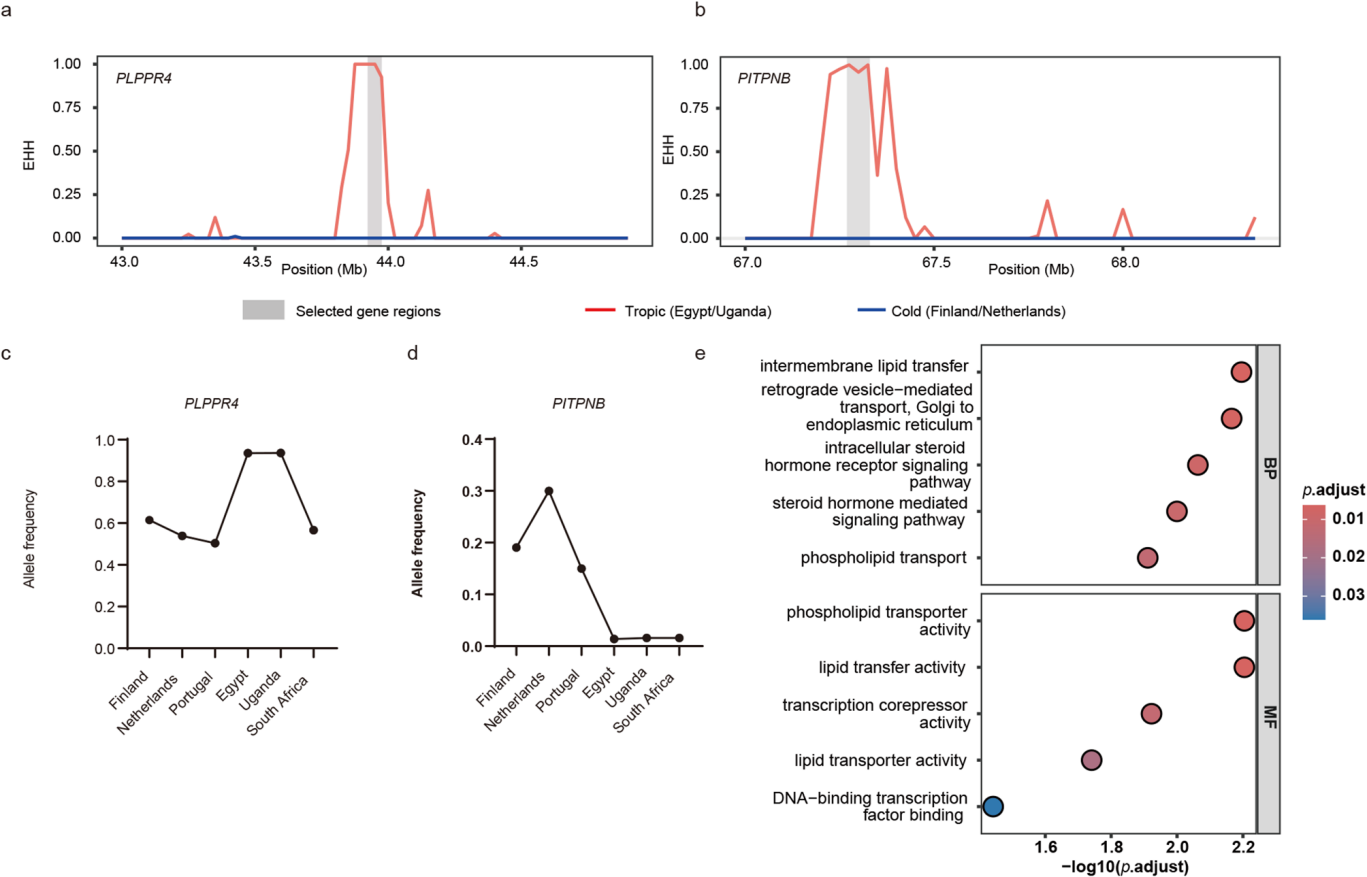
Annotation of candidate genes. **a** and **b** XP-EHH analyses of the most significant sweeps and surrounding regions on *PLPPR4* and *PITPNB* genes, comparing tropical (Uganda and Egypt) and colder (Finland and The Netherlands) zones by a non-overlapped 25 kb sliding window. The grey color highlights the regions of candidate genes. **c** and **d** The allele frequency of alternative alleles on the candidate genes of *PLPPR4* and *PITPNB*. **e** GO functional enrichment analysis for candidate genes identified within selective sweep regions. The GO analysis was divided into two categories: Biological Process (BP) and Molecular Function (MF). The point colors refer to the significance of the pathway terms (False discovery rate *p*-value < 0.05)

Among the candidate selective sweeps, the most significant sweeps included two candidate genes, *PLPPR4* and *PITPNB* (Fig. 5a). Compared to temperate zones, haplotype pattern analyses for *PLPPR4* and *PITPNB* in tropical zones showed relatively long haplotypes due to strong LD [24] (Fig. 5b and c, Supplementary material 9). The *θ*_π_ ratio (Fig. 5d and e), EHH values (Fig. 6a and b), and allele frequency (Fig. 6c and d) of selected gene regions between tropical HF populations and temperate zones exhibited reduced genetic diversity, increased haplotype homozygosity, and shifted alternative allele frequency in the selective sweeps for *PLPPR4* and *PITPNB*.Functional enrichment analysis using GO was performed for all overlapping genes (Fig. 6e). Detailed information for all overlapping regions (10 regions within 0.57 Mb) is shown in Supplementary material 9.

## Discussion

We analyzed WGS data from 30 HF cattle across six geographically district African and European countries and identified genomic regions, which may have been shaped by country-specific ecosystems since their introduction approximately a century ago. We also identified well-differentiated allele frequencies and candidate selection regions within these HF populations, including immune-response-, metabolic-, and organ-development-related genes. Last, we observed several candidate selective sweeps in tropical HF populations putatively linked to thermotolerance and phospholipid metabolic regulation. Together, these comprehensive genetic results support our hypothesis that the present HF genome might have been shaped by an early adaptation to local ecosystems in these countries.

Based on sporadic historical records, it is estimated that HF were initially imported to African and European countries around one century ago through the use of live animals and frozen semen [6, 7, 10, 12]. These cattle were introduced into diverse climates, ranging from the cold conditions of Finland in the mid-20th century to the hot, arid environment of Uganda in the early 20th century [5, 11]. Given these varied environmental conditions and the human breeding practices in different regions, we assume that today’s local HF genomes in various countries may have been shaped by a combination of artificial and natural selection. This hypothesis is supported by our PCA, hierarchical clustering, and ADMIXTURE analyses, which indicate that the genetic structure of HF cattle exhibits divergence across different ecosystems.

Genetic response to selection pressures can been linked to changes of allele frequencies [38]. By analyzing shifted allele frequencies at SNPs and hitchhiking regions, we identified several innate or adaptive immune-related genes in tropical and subtropical countries that show evidence of selection. For instance, genes such as *PGLYRP4*, *PGLYRP3*, and *PAG1* in Egypt; *CD84*, *SLAMF1* and *DYSF* in Portugal; and *LOC615223* in Uganda [39–41]. This may indicate early adaptation of HF cattle in these regions to combat infectious disease or parasites, which was also observed in genomes of Ugandan goats and African indigenous cattle breeds [15, 42]. We also identified several genes associated with development, such as in Egypt *LDB3* for muscle development and *ADAMTSL4* for pigment cell development [43], for Finland *TPRN* for hair development [44], *CCDC40* for reproduction [45], and *OR2AG1G* and *OR8B3* for sensory and olfactory functions [44, 46, 47]. Another candidate gene in Finland, *TBC1D16*, plays a role in membrane trafficking and molecule transport and is known to be dysregulated in obesity [48]. In South Africa, *VPS36* and *FOXO1*, related to membrane recruitment and catabolic processes, are pivotal in the transcriptional regulation of energy metabolism in relatively hot regions [49–51]. The HF population in Uganda shows evidence of possible selection at *R3HDM1*, a gene associated with meat quality and intramuscular fat content, which has been shown to be under positive selection in various cattle breeds (HF, Jersey, and Limousin) [52]. The above-mentioned data shed light into how specific environments may interact with development of the animal genome to cope with its challenges. In addition, these data also give good indication on how framer preferences towards specific traits might affect the decision of selecting frozen semen for artificial selection and shaping the genome of a certain breed.

Given HF cattle’s predisposition towards temperate regions [17, 18, 53], our study focused on identifying selective sweeps within the tropical regions (Egypt/Uganda). Hierarchical clustering and ADMIXTURE analyses also revealed that HF individuals from tropical regions were genetically distinct from those in colder regions. The most significant selective sweeps contained genes encoding the PLPPR4 and PITPNB proteins, which play a vital role in phospholipid metabolism [54, 55]. Phospholipids are associated with metabolism adaptation and have been used as heat stress biomarkers for dairy cattle [56]. Another significant region harboured the heat-tolerance gene *ZNF423*, which has been implicated in white adipose cell function [57, 58]. Another candidate gene, *ARFGEF3*, has been observed to exhibit regulation of insulin and glucagon secretion in Jersey under heat stress conditions [53, 59, 60]. These thermotolerance and metabolic sweeps support the hypothesis that the genetic foundation of HF cattle may have undergone early adaptation to tropical regions.

## Conclusions

HF cattle play a crucial role in the human-mediated global distribution of dairy breeds. Despite the complexity of ecosystems across African and European counties and the sporadic recording of precise time points for the extensive movement of HF cattle, our study expands the catalogue of genetic variants across distinct geographic regions. These findings highlight specific genomic regions and candidate genes that may have been subject to early selection pressures within HF populations, providing insights into the genetic basis of adaptation to environmental conditions in various countries.

## Materials and methods

### Sample collection and sequencing

Thirty HF cows were selected for this study, with five individuals from each of six countries: three African countries (Egypt, Uganda, and South Africa) and three European countries (Finland, the Netherlands, and Portugal). To minimize the impact of genetic relatedness, animals were carefully chosen to be unrelated for at least two generations. To reduce potential sampling bias and ensure broader genetic representation, cows from each country were sourced from 1 to 3 geographically distinct farms. This sampling strategy was designed to capture a representative genetic profile within each country while minimizing the influence of familial relationships on subsequent analyses.

These countries are located in a wide magnitude of geographic zones by latitude (Fig. 1a). Finland mostly belongs to the Boreal biogeographic zone, and northernmost part of Finland can be classified as humid continental climate and subarctic climate with cold, snowy winters and short, warm summers. The average annual temperature of Southern and Central Finland is approximately 3 °C (37 °F). The Netherlands lies in the temperate zone, with mean winter temperatures around 3 °C (37 °F) and mean summer temperatures about 17 °C (62.6 °F). Portugal experiences warm summers and moderate winters, with average temperatures ranging from 17 °C (62.6 °F) in January through March to 27 °C (80.6 °F) in the summer. Egypt has an arid desert climate, typically hot and sunny, with average high temperatures around 32 °C (89.6 °F) during the day and average lows of 23 °C (73.4 °F) at night. Uganda’s climate is largely tropical, with mean temperatures between 20 °C and 25 °C (68 °F and 77 °F). South Africa features both subtropical and temperate climate conditions, with temperatures ranging from 16 °C (61 °F) to 20 °C (68 °F).

These animals constituted a subset of the LEAP-Agri project OPTIBOV (https://subsites.wur.nl/en/optibov-project/about-optibov.htm). Blood samples were collected during the animals’ annual health inspections, conducted by licensed veterinarians. Prior to sample collection, written informed consent was obtained from each animal’s owner. Genomic DNA was extracted from EDTA-blood samples using the GENTRA Blood kit (Qiagen N.V.). The DNA quality and quantity were assessed using a Qubit fluorometer (Qiagen N.V.). DNA-sequence libraires were prepared by using the DNA Library Prep Kit (Illumina Inc., USA) and sequenced on the Illumina NovaSeq6000 platform (Illumina Inc., USA) with paired-end 150 bp reads. In addition, 42 HF samples were obtained from the 1000 Bull Genomes Project (Run7 version) [32, 61, 62] for population structure analyses.

### Short read pre-processing, variant calling, and filtering

The pre-processing of all raw sequencing data was performed using fastp v0.23.4 [63]. This involved several steps: trimming adapter sequences, correcting mismatched bases in overlaps of paired-end reads, and removing duplicate sequences and reads of suboptimal quality. To ensure data reliability, reads with an average quality score below 30 and those shorter than 36 bases were discarded.

Subsequently, the clean reads were mapped to the bovine reference genome (assembly version ARS-UCD1.2) using Bwa mem2-v2.2.1 [64]. The resulting SAM/BAM files from the mapping process underwent further processing, which included marking duplicate reads with samblaster v0.1.26 [65], sorting by coordinates using Samtools v1.14 [66], and generating a mapping quality report with QualiMap v2.0 [67]. The sorted BAM files were then utilized for variant calling.

SNP calling was performed following the freebayes (v1.3.1) pipeline “population variants calling” on population samples followed by joint genotype [68]. During the variants calling steps, several filtration and statistical analyses were considered: variants with a Phred-scaled probability less than 20 and a coverage depth of less than 4 were removed using vcffilter/vcflib (v0.00.2019.07.10) [69]. For final quality control, SNPs with a missing genotype rate greater than 5% and MAF less than 5% across the samples were filtered out using VCFtools (v0.1.16) [70]. By setting a MAF threshold 5%, we aim to include only those alleles observed more than twice, thereby reducing the likelihood of false-positive results in our sample size. The final set of variants was annotated using the Variant Effect Predictor (VEP) v111, a comprehensive tool for annotating variants found in genomic regions [71].

### Analysis of the population structure and individual ancestry

To assess the genetic population structure of HF across different countries, PCA was performed in PLINK v1.9 [72], targeting the 29 chromosomes (autosomes). The parameters applied for biallelic variants with: --maf 0.05, --geno 0.05, and --indep-pairwise 50 10 0.2 --pca 4. The PCA plots were visualized using the R package ggplot2 [73].

To estimate the genetic clustering of HF populations across different countries, hierarchical clustering using the Ward method was employed [74]. This analysis was performed in PLINK v1.9 [72], targeting the 29 autosomes, with the parameters: --maf 0.05, --geno 0.05 --indep-pairwise 50 10 0.2, and --distance-matrix. The hierarchical clustering plot was visualized using the R package stats [75].

ADMIXTURE v1.3.0 software [76] was used to estimate individual ancestry and the possibility of subpopulation structure. The optimal number of ancestral populations was identified based on the lowest CV. ADMIXTURE analyses considered *K*-values ranging from 1 to 5 on 29 autosomes with biallelic variants, using parameters consistent with those applied in the PCA: --maf 0.05, --geno 0.05, and --indep-pairwise 50 10 0.2. The results of the ADMIXTURE analyses were visualized by the ggplot2 package in R [73].

### Estimation of allele frequency divergence between populations

To pinpoint SNPs that are potentially advantageous under selection between HF populations across six countries, allele frequency (AF) divergence was used as a criterion. Specifically, SNPs with an AF ≥ 0.6 in any one country (with at least three individuals) and an AF < 0.3 in the remaining five countries were considered. This cutoff reflects a moderate but biologically meaningful threshold, recognizing the limited evolutionary timeframe (up to 100 years) since the introduction of HF cattle into these countries. It captures the early-to-intermediate phases of allele fixation to emphasize potential regional selection signals [77]. After identifying these potential regional SNPs, the genetic relationships of the corresponding SNPs within each country were examined by a PCA. This analysis was conducted by PLINK v1.9 with parameters: --maf 0.05, --geno 0.05, --indep-pairwise 50 10 0.2, and --pca4 [72]. The PCA plot was visualized by R package ggplot2 [73].

### Detection of genome-wide selection signatures

The process of identifying genome-wide selection signatures within each country was conducted by *F*st [78]. The *F*st method measures genetic differentiation between populations, identifying loci and nearby loci with significant divergence in allele frequencies. Despite limited sample sizes, *F*st estimates of genetic differentiation remain relatively reliable and do not require large sample sizes (as small as *n* = 4–6) when using a substantial number of SNP markers (> 1,000) with unrelated samples and independent loci [36]. This makes *F*st an effective tool for detecting selection signatures in our study, where unrelated sample sizes are constrained but SNP counts are extensive.

The initial phase of identifying selection signatures involved excluding variants with a genotype missing rate over 5% and missing counts above 2. Subsequently, sliding windows were employed to scan the filtered variants using a 50 kb window size with a 25 kb step size, conducted with VCFtools v4.0 [70]. Windows with the top 0.1% *F*st values were identified as significant genomic regions. To avoid false positives, each peak was required to have at least three continuous windows above the significance threshold (top 0.1% *F*st values). Manhattan plots were visualized using the R package qqman [79].

### Detection of selective sweeps in tropical cattle populations from Uganda and Egypt

To detect selective sweeps between samples from tropical (Uganda and Egypt) and colder (Finland and The Netherlands) zones, we integrated three approaches: *F*st, *θ*_π (tropic/temperate)_ ratio, and XP-EHH [15, 80, 81].

Initially, variants with a genotype missing rate over 5% and missing counts above 2 were excluded. Then. sliding windows of *F*st, genetic diversity (*θ*_π (tropic/temperate)_ ratio), and XP-EHH were employed to scan the selective outliers using a non-overlapping 25 kb window size by VCFtools v4.0 [70] and selscan v2.0 [82]. The overlapped sweeps (top 1% values of *F*st, *θ*_π (tropic/temperate)_ ratio, and XP-EHH more than 2) were identified as significant genomic regions for allele frequency differentiation, reduced genetic diversity, and increased haplotype homozygosity in tropical zones [83, 84]. Manhattan plots were visualized using the R package qqman v0.1.9 [79]. The results of the haplotype structure were visualized using the ggplot2 v3.5.2 package in R [73].

### Annotation and functional enrichment analyses

All variants were annotated using the VEP v111, a comprehensive tool for characterizing genomic variants [71]. Detailed annotations and the distribution of variants are provided in Supplementary Material 2. SNP density was calculated using VCFtools v4.0 (--SNPdensity 1000000) [70] and visualized with TBtools-II v2.007 [85]. GO enrichment analyses were performed using KOBAS v3.068 [86]. Only GO terms with a false discovery rate (FDR): adjusted *p*-value less than 0.05 were considered significantly enriched. QTL enrichment analyses were conducted using GALLO v1.5 [87], with trait associations retrieved from the Animal QTLdb (https://www.animalgenome.org/cgi-bin/QTLdb/index). Traits with FDR-adjusted *p*-values < 0.05 were considered significantly enriched. All plots were generated using the ggplot2 v3.5.2 package in R [73].

## Supplementary Information

Supplementary Material 1. Detailed information on samples available in ENA and mean coverages of mapping data. (XLSX format). Supplementary Material 2. Table S1: Distribution of variants in raw.vcf file. Table S2: Summary of high-quality SNPs and SNP distribution on each chromosome (30 HF). Table S3: Summary of SNP types. Table S4: Summary of coding region SNPs. Table S5: Summary of prediction effects of nonsynonymous/missense SNPs (SIFT). SIFT predicts whether an amino acid substitution affects protein function based on sequence homology and the physical properties of amino acids. All variants were collected from three African countries (Egypt, South Africa, and Uganda) and three European countries (Finland, The Netherlands, and Portugal). (DOCX format). Supplementary Material 3. Distribution of variant classes and number of variants in 72 HF cattle samples from six African and European countries and 42 previously published HF genomes from 1000 Bull Project. (DOCX format). Supplementary Material 4. Figure S1: ADMIXTURE and Cross-validation error plot. a. Cross-validation error plot: The optimal cross-validation error (CV) at *K* = 1 from ADMIXTURE analysis. b. ADMIXTURE plot with *K* = 1-3: Columns correspond to individual animals. *K* = 1 indicates the most likely number of clusters.(DOCX format). Supplementary Material 5. This table provides annotations for gene-coding SNPs with diverged allele frequencies across each country. (XLSX format). Supplementary Material 6. Additional information on significant regions (*F*st 0.1%) and associated genes. (XLSX format). Supplementary Material 7. Enrichment of QTL traits and categories on significant regions (*F*st 0.1%) across each country. (DOCX format). Supplementary Material 8. Additional information on separately significant outliers (top 1% for *F*st; top 1% for *θ*_π_ ratio; XP-EHH > 2) and associated genes. (XLSX format). Supplementary Material 9. Additional information on overlapping significant regions (top 1% for *F*st & top 1% for *θ*_π_ ratio & XP-EHH> 2) and associated genes. (XLSX format).

## Data Availability

The raw full-length sequencing data (in FASTQ format) have been submitted to the European Nucleotide Archive (ENA) under the project accession number PRJEB76602. The specific accession numbers for the FASTQ files are ERR13293989 to ERR13293990, ERR13296442 to ERR13296449, ERR13299691 to ERR13299696, and ERR13300210 to ERR13300233.

## Abbreviations

ABC: ATP-Binding Cassette
AF: Allele Frequencies
AI: Artificial Insemination
BTA: Bos Taurus Autosome
BoLA: Bovine Leukocyte Antigen
FDR: False Discovery Rate
Fst: F-statistic
Gb: Gigabyte
GO: Gene Ontology
HF: Holstein Friesian
LD: Linkage Disequilibrium
MAF: Minor Allele Frequency
Mb: Megabase
MHC: Major Histocompatibility Complex
PCA: Principal Components Analysis
SIFT: Sorting Intolerant From Tolerant
SNP: Single Nucleotide Polymorphism
θ_π_ratio: Nucleotide Diversity Analysis
VEP: Variant Effect Predictor
XP-EHH: Cross Population Extended Haplotype Homozygosity
