# Supplementary material for: Evidence of early genomic selection in Holstein Friesian across African and European ecosystems
Source: BMC Genomics. 2025 Jul 1;26:615. doi: 10.1186/s12864-025-11828-y (PMC12211335; doi:10.1186/s12864-025-11828-y)
Supplement: Supplementary file 1 — Supplementary Material 1. Detailed information on samples available in ENA and mean coverages of mapping data. (XLSX format). Supplementary Material 2. Table S1: Distribution of variants in raw.vcf file. Table S2: Summary of high-quality SNPs and SNP distribution on each chromosome (30 HF). Table S3: Summary of SNP types. Table S4: Summary of coding region SNPs. Table S5: Summary of prediction effects of nonsynonymous/missense SNPs (SIFT). SIFT predicts whether an amino acid substitution affects protein function based on sequence homology and the physical properties of amino acids. All variants were collected from three African countries (Egypt, South Africa, and Uganda) and three European countries (Finland, The Netherlands, and Portugal). (DOCX format). Supplementary Material 3. Distribution of variant classes and number of variants in 72 HF cattle samples from six African and European countries and 42 previously published HF genomes from 1000 Bull Project. (DOCX format). Supplementary Material 4. Figure S1: ADMIXTURE and Cross-validation error plot. a. Cross-validation error plot: The optimal cross-validation error (CV) at K = 1 from ADMIXTURE analysis. b. ADMIXTURE plot with K = 1-3: Columns correspond to individual animals. K = 1 indicates the most likely number of clusters.(DOCX format). Supplementary Material 5. This table provides annotations for gene-coding SNPs with diverged allele frequencies across each country. (XLSX format). Supplementary Material 6. Additional information on significant regions (Fst 0.1%) and associated genes. (XLSX format). Supplementary Material 7. Enrichment of QTL traits and categories on significant regions (Fst 0.1%) across each country. (DOCX format). Supplementary Material 8. Additional information on separately significant outliers (top 1% for Fst; top 1% for θπ ratio; XP-EHH > 2) and associated genes. (XLSX format). Supplementary Material 9. Additional information on overlapping significant regions (top 1% for Fst & top 1% f [file 12864_2025_11828_MOESM1_ESM.zip › Additional files/Supplementary Material 3.docx]

This file was produced by bcftools stats (1.9+htslib-1.9) and can be plotted using plot-vcfstats.

# The command line was: bcftools stats 72HF.vcf.gz

#

# Definition of sets:

# ID [2]id [3]tab-separated file names

ID 0 1000HF+myhf.2.8.vcf.gz

# SN, Summary numbers:

# number of records .. number of data rows in the VCF

# number of no-ALTs .. reference-only sites, ALT is either "." or identical to REF

# number of SNPs .. number of rows with a SNP

# number of MNPs .. number of rows with a MNP, such as CC>TT

# number of indels .. number of rows with an indel

# number of others .. number of rows with other type, for example a symbolic allele or

# a complex substitution, such as ACT>TCGA

# number of multiallelic sites .. number of rows with multiple alternate alleles

# number of multiallelic SNP sites .. number of rows with multiple alternate alleles, all SNPs

#

# Note that rows containing multiple types will be counted multiple times, in each

# counter. For example, a row with a SNP and an indel increments both the SNP and

# the indel counter.

#

# SN [2]id [3]key [4]value

SN 0 number of samples: 72

SN 0 number of records: 148600272

SN 0 number of no-ALTs: 0

SN 0 number of SNPs: 132118261

SN 0 number of MNPs: 0

SN 0 number of indels: 19015712

SN 0 number of others: 2074937

SN 0 number of multiallelic sites: 14516284

SN 0 number of multiallelic SNP sites: 8066833
