# Supplementary material for: Evidence of early genomic selection in Holstein Friesian across African and European ecosystems
Source: BMC Genomics. 2025 Jul 1;26:615. doi: 10.1186/s12864-025-11828-y (PMC12211335; doi:10.1186/s12864-025-11828-y)
Supplement: Supplementary file 1 — Supplementary Material 1. Detailed information on samples available in ENA and mean coverages of mapping data. (XLSX format). Supplementary Material 2. Table S1: Distribution of variants in raw.vcf file. Table S2: Summary of high-quality SNPs and SNP distribution on each chromosome (30 HF). Table S3: Summary of SNP types. Table S4: Summary of coding region SNPs. Table S5: Summary of prediction effects of nonsynonymous/missense SNPs (SIFT). SIFT predicts whether an amino acid substitution affects protein function based on sequence homology and the physical properties of amino acids. All variants were collected from three African countries (Egypt, South Africa, and Uganda) and three European countries (Finland, The Netherlands, and Portugal). (DOCX format). Supplementary Material 3. Distribution of variant classes and number of variants in 72 HF cattle samples from six African and European countries and 42 previously published HF genomes from 1000 Bull Project. (DOCX format). Supplementary Material 4. Figure S1: ADMIXTURE and Cross-validation error plot. a. Cross-validation error plot: The optimal cross-validation error (CV) at K = 1 from ADMIXTURE analysis. b. ADMIXTURE plot with K = 1-3: Columns correspond to individual animals. K = 1 indicates the most likely number of clusters.(DOCX format). Supplementary Material 5. This table provides annotations for gene-coding SNPs with diverged allele frequencies across each country. (XLSX format). Supplementary Material 6. Additional information on significant regions (Fst 0.1%) and associated genes. (XLSX format). Supplementary Material 7. Enrichment of QTL traits and categories on significant regions (Fst 0.1%) across each country. (DOCX format). Supplementary Material 8. Additional information on separately significant outliers (top 1% for Fst; top 1% for θπ ratio; XP-EHH > 2) and associated genes. (XLSX format). Supplementary Material 9. Additional information on overlapping significant regions (top 1% for Fst & top 1% f [file 12864_2025_11828_MOESM1_ESM.zip › Additional files/Supplementary Material 4.docx]

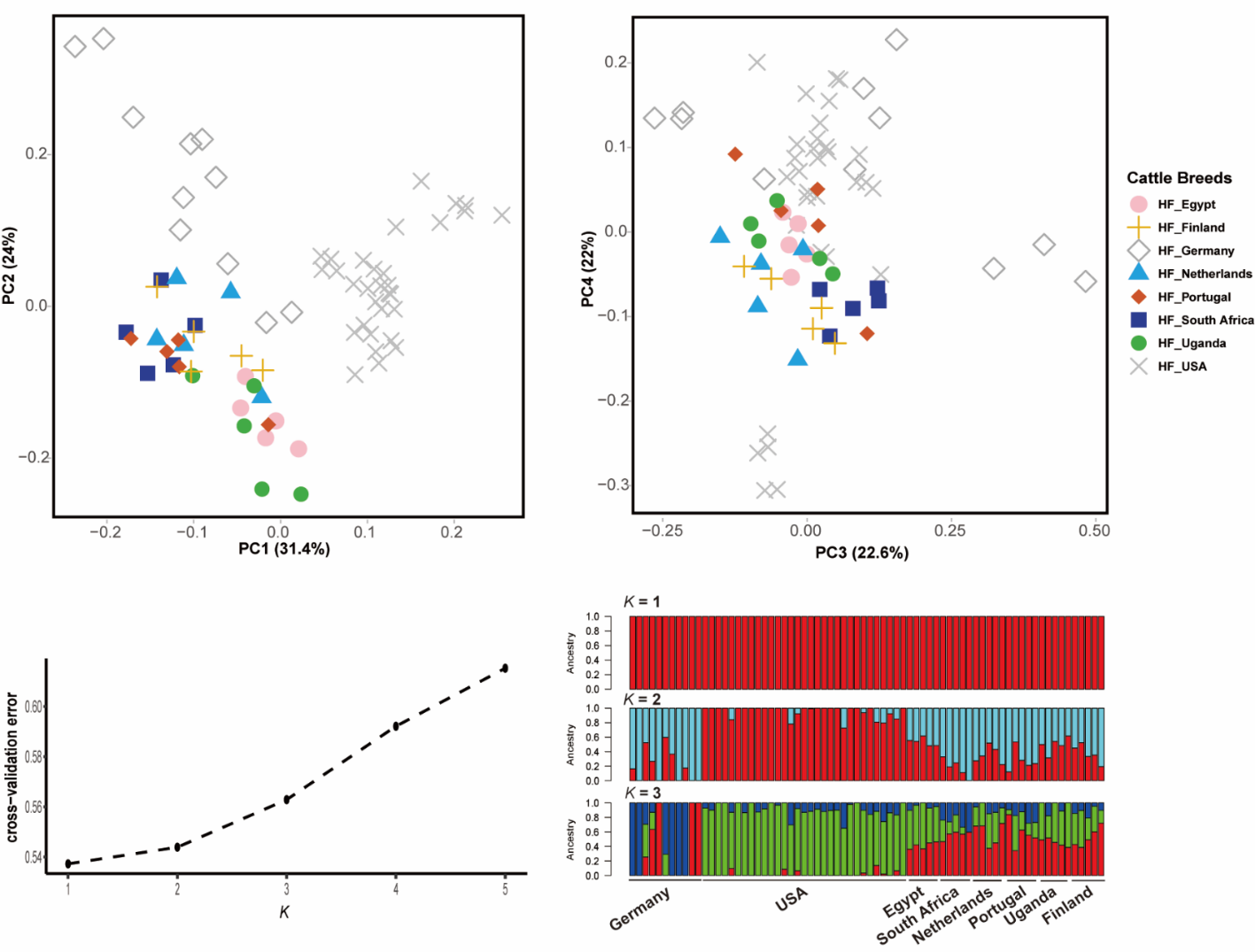
**Figure S1:** To identify the genetic background of Holstein Friesian (HF) cattle, 30 HF genomes were combined with 42 previously published HF genomes from the 1000 Bull Genomes Project.

b

a

**a**. Cross-validation error plot: The optimal cross-validation error (CV) at *K* = 1 from ADMIXTURE analysis.

**b**. ADMIXTURE plot with *K* = 1-3: Columns correspond to individual animals. *K* = 1 indicates the most likely number of clusters.
